# Characterization of an Invertase with pH Tolerance and Truncation of Its N-Terminal to Shift Optimum Activity toward Neutral pH

**DOI:** 10.1371/journal.pone.0062306

**Published:** 2013-04-19

**Authors:** Liqin Du, Hao Pang, Zilong Wang, Jian Lu, Yutuo Wei, Ribo Huang

**Affiliations:** 1 State Key Laboratory for Conservation and Utilization of Subtropical Agro-bioresources, Guangxi University, Nanning, Guangxi, China; 2 National Engineering Research Center for Non-Food Biorefinery, Guangxi Academy of Sciences, Nanning, Guangxi, China; Aligarh Muslim University, India

## Abstract

Most invertases identified to date have optimal activity at acidic pH, and are intolerant to neutral or alkaline environments. Here, an acid invertase named uninv2 is described. Uninv2 contained 586 amino acids, with a 100 amino acids N-terminal domain, a catalytic domain and a C-terminal domain. With sucrose as the substrate, uninv2 activity was optimal at pH 4.5 and at 45°C. Removal of N-terminal domain of uninv2 has shifted the optimum pH to 6.0 while retaining its optimum temperaure at 45°C. Both uninv2 and the truncated enzyme retained highly stable at neutral pH at 37°C, and they were stable at their optimum pH at 4°C for as long as 30 days. These characteristics make them far superior to invertase from *Saccharomyces cerevisiae*, which is mostly used as industrial enzyme.

## Introduction

β-Fructofuranosidases (EC 3.2.1.26) are enzymes that are capable of hydrolyzing substrates with terminal fructosyl. Most β-fructofuranosidases have been shown to hydrolyze sucrose to release glucose and fructose and to possess fructosyltransferase activity for the synthesis of short-chain fructooligosaccharides [1]. Based on the hydrolysis substrates, β-fructofuranosidases can be given different names, invertase for sucrose hydrolysis, and inulinase and β-fructosidase for inulin hydrolysis [2]. The ratios of β-fructofuranosidase activities for sucrose and inulin vary widely, it has been shown that β-fructofuranosidase from some *Bifidobacteria* could hydrolyze fructooligosaccharides faster than sucrose [2].

Invertase activity catalyzes the hydrolysis of sucrose to produce a mixture of fructose and glucose (inverted sugar syrup). Invertase is important for the industrial hydrolysis of sucrose, which is a sustainable carbohydrate resource used in food and fermentation process. In the food industry, the use of invertases ensures that the confectionery products remain fresh and soft after a long period of storage [3]. Invert sugar, the invertase hydrolysed product of sucrose, consists of an equimolar mixture of glucose and fructose. It is sweeter than sucrose and has a lower crystallinity than sucrose at higher concentrations [4]. This mixture has similar properties to high fructose syrup from starch sources, and can be used as an alternative to the same. For these reasons, invertases are used widely in various industrial food applications. In the alcohol industry, invertase activity is used in the fermentation of cane molasses into ethanol where it is required for its ability to hydrolyze sucrose under the inhibitor conditions existing in molasses [5]. Other uses of these enzymes include the production of plasticizing agents in cosmetics, drug and paper industries, and as enzyme electrodes in bioelectronic applications [6], [7]. Recently, enzyme electrode sensors have been the target of intense research, and new methods allow this technology to be used more broadly [7], [8]. In enzyme biosensors, the sucrose hydrolysis activity of invertase is used as a signal, and it is preferable that this enzyme is catalytically active under neutral pH conditions and does not participate in adverse or side reactions such as transferase activity [9].

To date, the most well studied and readily available invertase is the acid invertases derived from *Saccharomyces cerevisiae*. The neutral invertases, which are mainly found in plants, have neutral pH activity and many appear to use sucrose as their sole substrate [10]. Not very much is known about neutral/alkaline invertases at the native protein level due to difficulties in their purification and their low, unstable enzymatic activities [11], although some studies have cloned and expressed neutral invertases in *Escherichia coli* [10], [12], [13]. Using immobilized enzyme technologies the stabilization of the invertase has been achieved under extreme conditions [14]. Such technologies may help to shift the optimum pH of the invertase and prevent the formation of oligosaccharides by the transferase activity that is often also associated with the soluble enzyme [15].

We previously constructed a metagenomic library from sucrose rich soils and screened for sucrose-degrading enzymes [16]. In the previous work, invertase uninv was found and characterized, it had an optimum pH of 6.5 and an optimum temperature of 50°C toward sucrose substrate. In the present study, an invertase gene was subcloned and characterized from this metagenomic library. This recombinant invertase showed a strong activity and pH stability. Furthermore, we engineered this gene to adjust the optimum pH of the invertase from acidic to near neutral pH.

## Materials and Methods

### Bacterial Strains and Materials

*E. coli* strain XL1-blue and EPI300 were purchased from Takara Bio Inc. (Dalian, China), and Epicentre Inc. (Madison, WI), respectively. Vector pCC1FOS was purchased from Epicentre Inc., vector pUC19 and pSE380 were purchased from Invitrogen Inc. (San Diego, CA). The chemicals were purchased from Sigma-Aldrich Chemical Co. (St. Louis, MO). The enzymes including restriction endonucleases and DNA ligase, and PCR primers were purchased from Takara Bio Inc.

### Library Screening and Gene Cloning

We previously constructed a metagenomic library from sucrose rich soils [16]. Clones of the fosmid DNA library were screened on M9 basic medium containing 10 mg l^−1^ yeast extract, with 10 g l^−1^ sucrose as the sole carbon source. The library, grown on Luria agar (LA) medium, was transferred to the M9 plate and incubated at 37°C for 50 h. The clones that grew most rapidly were selected for further enzyme activity assessment. The chosen clones were lysed by sonication and the sucrose hydrolysis activity of the lysate was examined using 3′,5′-dinitrosalicylic acid (DNS) [17]. The plasmid that harbored the positive clone was extracted, digested with *Bam*HI, and the fragments were subcloned into a pUC19 vector. The sub-cloned fragments were then sequenced from both ends by Takara Bio Inc.

### Sequence Analysis of Uninv2

**Figure 1:**
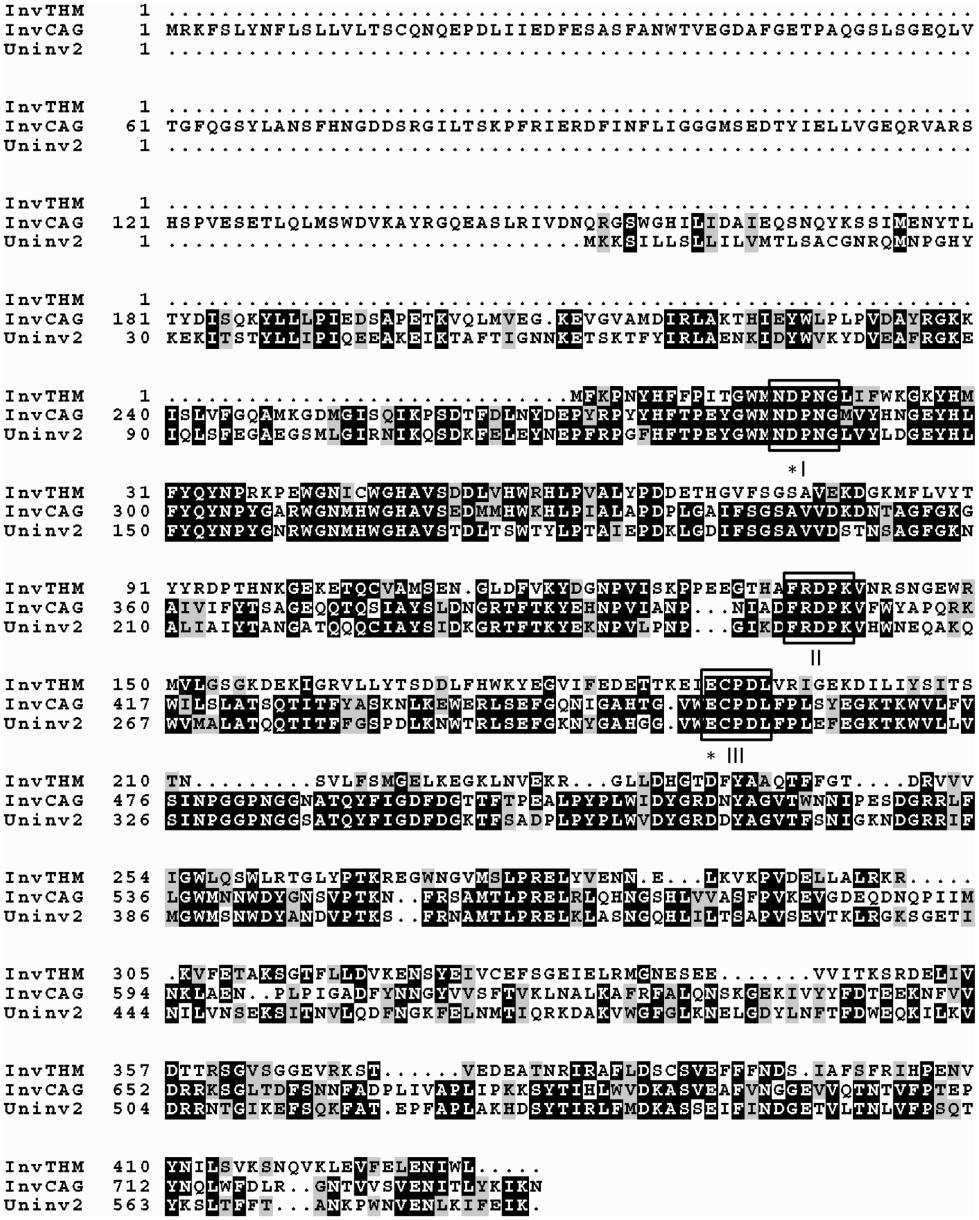
Sequences alignment of uninv2, the invertase from *T. maritima* and the invertase2 from *C. gingivalis*. The sequences were identified as follows: uninv2 (HQ267532), invTHM, invertase from *T. maritima* (CAA04518), invCAG, invertase2 from *C. gingivalis* (EEK13630). The boxes (I–III) indicate the conserved NDPNG, FRDP and ECP motifs of the glycoside hydrolase family 32 invertases. * mark the catalytic active residues and general acid/base. The alignment was performed with MUSCLE and the figure was produced with BOXSHADE.

Sequence similarity searches were performed using BLAST tools from the National Center for Biotechnology Information (www.ncbi.nlm.nih.gov). The signal peptide was predicted using signalP (http://www.cbs.dtu.dk/services/SignalP/). Protein domain analysis was performed with the Simple Modular Architecture Research Tool (SMART) on the SMART server (http://smart.embl-heidelberg.de). Alignment analysis was performed with MUSCLE (http://www.ebi.ac.uk/Tools/muscle/), and Fig. 1 was produced with BOXSHADE (http://www.ch.embnet.org/software/BOX_form.html).

### Cloning and Expression of the *uninv2* Gene

First, *uninv2* was amplified with the following primers: sense primer F1, 5′ -CACTCATGATGCACCACCACCACCACCACAATAGACAGATGAATCCAGGTC -3′ (containing a *Pag*I site and a 6×His tag at the 5′ end) and anti-sense primer R1, 5′ - CACCTGCAGACGATGATTTCACAGATGCAAGC -3′ (containing a *Pst*I site at the 5′ end) by PCR. Amplified DNA was digested with *Pag*I and *Pst*I and sub-cloned into a pSE380 expression vector (Invitrogen) digested with *Nco*I and *Pst*I. The expression construct was transformed into *E. coli* XL1-blue. The transformants were incubated at 37°C until an OD_600_ of 0.6 was reached. Then 1 mM IPTG was added to the broth and incubation was continued at 37°C for a further 10 h to induce expression of the enzyme. The recombinant protein was purified with nickel-nitrilotriacetic acid chromatography (Qiagen, Hilden, Germany) according to the manufacturer’s instructions. The purified protein was then passed through a 30 kDa ultra-filter membrane (Whatman, Kent, UK) to dialysis the protein. The purified protein was analyzed with sodium dodecyl sulfate–polyacrylamide gel electrophoresis (SDS–PAGE) and matrix assisted laser desorption ionization mass spectrometry (MALDI-MS). The MALDI-MS analysis was performed on the Bruker Autoflex II MALDI-TOF/TOF System (Bruker, Bremen, Germany).

### Truncation of *Uninv2*

*Uninv2* was truncated to delete the DNA encoding the 100 amino acids N-terminal domain by PCR with the sense primer F2 5′- CACTCATGATGCACCACCACCACCACCACTTTCGACCTGGATTTCATTTC-3′ (containing a *Pag*I site and a 6×His tag at the 5′ end, this primer started from nucleotide 361 in the *uninv2* gene, relative to the ATG start codon), and anti-sense primer R1. The truncated uninv2 gene was sequenced three times to confirm that the sequence was correct. The truncated *uninv2* was named *M-inv2*, and the M-inv2 protein was expressed and purified as described above.

### Determination of Enzyme Activity and Kinetic Parameters

Sucrose hydrolysis reactions involved incubation at 45°C for 30 min of reaction mixtures containing the purified protein and 10 g l^−1^ sucrose in a volume of 0.5 ml at the optimal pH. The amount of proteins (uninv2 and M-inv2) used in a single reaction was 0.1 µg. The activity was quantified by measuring the reducing sugars generated (as d-glucose equivalents) with DNS reagent. One enzyme unit (U) corresponded to 1 µmol of glucose equivalent released from the reaction per min under the above conditions. The pH dependence of the enzymes’ activity was measured between pH 2.5 and 7.5 using 50 mM glycine-HCl buffer (pH 2.5–3.5), HAC-NaAC buffer (pH 3.5–6.0), or Na phosphate buffer (pH 6.0–8.0), at 37°C. The temperature dependence of the enzymes’ activity was measured between 20°C and 60°C at the optimal pH. For comparing the pH stability of the enzymes, the protein was placed at 37°C, and after different time intervals, aliquots were removed for enzyme activity assays. For determination of storage stability, the protein was placed at 4°C or 37°C, and the pH of the storage buffer was 6.0 for uninv [16], and 5.0 for uninv2 and M-inv2.

The activity of the enzymes for alternative substrates was also assayed. Sucrose, inulin, raffinose, 1-kestose and nystose were tested at 100 mM under the same conditions as those described above and the incubation time was 12 h, except that sucrose was only incubated for 5 min. The products formed were analyzed by high performance liquid chromatography (HPLC) using a 4.6 × 250 mm carbohydrate cartridge column on a Waters 2695 system equipped with an evaporative light scattering detector. Acetonitrile/water (70∶30) was used as the solvent with a flow rate of 1 ml min^−1^.

## Results and Discussion

### Library Screening and Gene Cloning

A Fosmid library containing about 100,000 clones was constructed with the soil sample from the sugar refinery as we described before [16]. A cluster of 5,000 clones from this library was screened for the target activity. Eight clones grew significantly faster on M9 (with sucrose) plates, one of which showed significant activity against sucrose in neutral pH buffer (pH 6.5–8.0, sodium-phosphate buffer) [16]. The other clones showed activity in the acid pH (<6.0) range. One clone showed the highest activity at acid pH (pH 4.5), and it was named 3–7. A subclone 3-7-s5 derived from 3–7 had activity toward sucrose. This subclone contained a 6,930 bp insert DNA, sequence analysis showed that there were three ORFs in the insert DNA: a putative transmembrane sugar transporter, a putative two-component system sensor histidine kinase/response regulator, and an enzyme that showed homology to an invertase 2 from *Capnocytophaga gingivalis* ATCC 33624 as described in GenBank (accession no. EEK13630). The third ORF, which was deemed to be the most likely sugar-degrading enzyme candidate, was chosen for further analysis. This ORF was named *uninv2*. The sequence of *uninv2* was deposited in GenBank with the NCBI under accession number HQ267532.

### Molecular Analysis of Uninv2

The putative protein encoded by *uninv2*, namely uninv2, contained 586 amino acids and the calculated molecular mass is 66.7 kDa. SignalP predicted that uninv2 contained a signal peptide at the N terminus. SMART analysis also showed that uninv2 contained a signal peptide (1–20 amino acids long), as well as a glycoside hydrolase family 32 domain (126 aa–549 aa).

Interestingly, uninv2 had a additional 120 amino acids domain present at the N-terminus comparing with invertase from *Thermotoga maritima* (Fig. 1). Only a few sucrose-hydrolyzsed enzymes contain an N-terminal domain of unknown function (Fig. S1). This N-terminal domain of uninv2 had no similarity to any known protein sequence in the GenBank. The remaining uninv2 sequence was most similar to invertase 2 from *C. gingivalis* ATCC 33624, according to BlastX analysis. Comparison of the uninv2 sequence with related glycoside hydrolase family 32 sucrose-degrading enzymes revealed the presence of several conserved regions in uninv2 (Fig. 1). The conserved NDPNG, FRDP and ECP motifs of the glycoside hydrolase family 32 invertases were all present in uninv2 [18]. This indicated that uninv2 was closely related to invertases from glycoside hydrolase family 32.

### Expression and Purification of Uninv2

**Figure 2:**
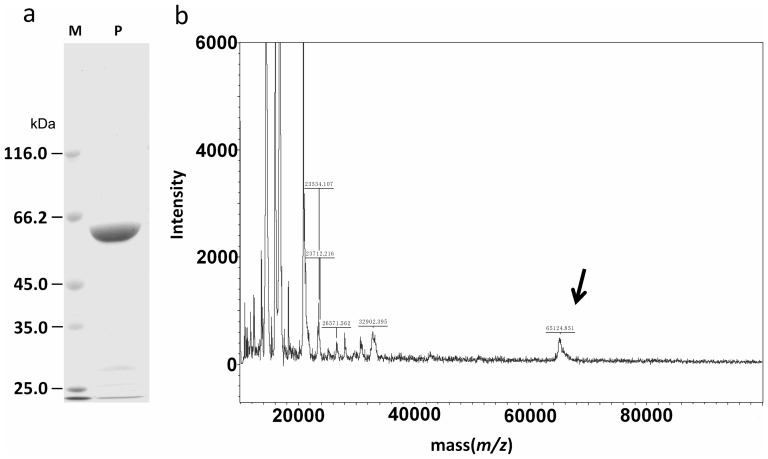
Analysis of purified recombinant uninv2. (a) SDS–PAGE analysis of uninv2 protein stained with coomassie blue. Lane M, markers for molecular size (kDa); Lane P, protein sample. The uninv2 is at 65 kDa by SDS-PAGE. (b) MALDI MS analysis of recombinant uninv2, the arrows indicate the target protein peaks.

The recombinant protein was over-expressed in *E. coli*, and one-step purification was performed with Ni–NTA chromatography. The purified uninv2 was homogeneous, giving a single and clean band at approximately 65 kDa on an SDS–PAGE gel (Fig. 2a). The time of flight mass spectrometry (TOF-MS) analysis showed a 65 kDa peak which corresponded to the deduced molecular weight of the *uninv2* gene product (Fig. 2b).

### Enzymatic Properties of Uninv2

**Figure 3:**
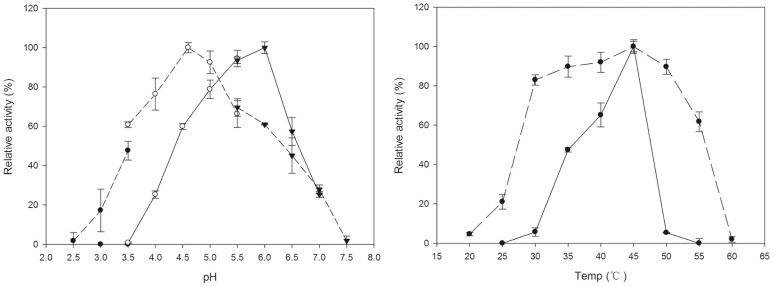
Effects of pH and temperature on the activity of recombinant uninv2 (dashed line) and M-inv2 (solid line). (a) Effect of pH on enzymatic activity, measured in 50 mM glycine-HCl buffer (pH 2.5–3.5, closed circles), HAC-NaAC buffer (pH 3.5–6.0, open circles) and Na phosphate buffer (pH 6.0–8.0, triangles) at 37°C for 30 min, the sucrose was 10 g l^−1^ in a volume of 0.5 ml. Enzyme activity is shown as the specific activity: one unit represents 1 µmol of glucose released from the reaction per min per mg protein. (b) Effect of temperature on the activity of uninv2 (dashed line) and M-inv2 (solid line). The temperature dependence of the enzymes’ activity was measured between 20°C and 60°C under the optimum pH condition (pH 4.5 or 6.0 for uninv2 and M-inv2, respectively). The error bars represent the standard deviation of triplicate measurements.

Uninv2 was active at pH 2.5–7.5, with an optimum pH of 4.5. It retained 50% of its activity between pH 3.5 and 6.0 (Fig. 3a). Uninv2 was found to be catalytically active between 20°C and 60°C, and had an optimum temperature of 45°C (Fig. 3b).

**Figure 4:**
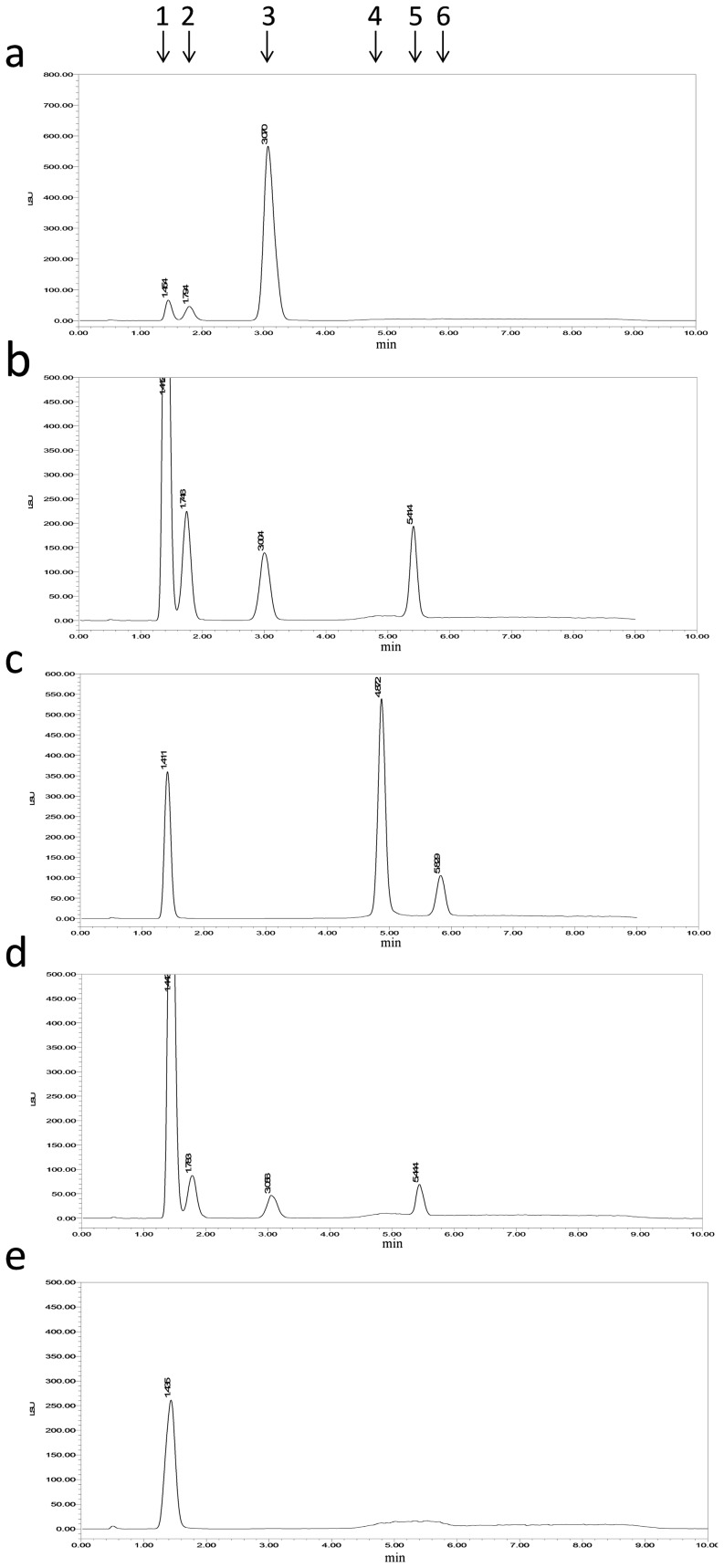
Substrate profiles of uninv2. Substrates: 100 mM sucrose (a), 1-kestose (b), raffinose (c), nystose (d) and inulin (e) were hydrolyzed by uninv2. All the reactions were performed in a volume of 0.5 ml pH 4.5 HAC-NaAC buffer at 45°C for 12 h, except that sucrose was only incubated for 5 min. Peak numbers show the retention times for fructose (1), glucose (2), sucrose (3), melibiose (4), 1-kestose (5) and raffinose (6).

Uninv2 was found to be capable of hydrolyzing sucrose, 1-kestose, raffinose, nystose and inulin. Sucrose was hydrolyzed to fructose and glucose; 1-Kestose was hydrolyzed to fructose, glucose and sucrose; raffinose was hydrolyzed to fructose and melibiose; nystose was hydrolyzed to fructose, glucose, and sucrose; and inulin was hydrolyzed to fructose (Fig. 4). No transglycosylation activity was detected by HPLC analysis of the reaction mixtures.

The optimum pH of uninv2 was similar to most acid invertases that have been reported to date, which have activity in the range of pH 3.5 to 6.0 and an optimum near pH 4.5 [3], [4]. Therefore, uninv2 can be classified as an acid invertase.

Uninv2 can hydrolyze sucrose, 1-kestose, raffinose, nystose and inulin. The hydrolysis of low molecular weight fructose-containing oligosaccharides is characteristic of invertases of the GH 32 family [19]. Since uninv2 released fructose from fructose linked oligosaccharides, for example nystose, and it can hydrolyze inulin to release fructose, it can be concluded that uninv2 is an exo-glycosidase. In contrast to most invertases, uninv2 can hydrolyze inulin, which is a poor substrate for acid invertases of the GH32 family [10], [19].

### Truncation of Uninv2: To Shift Optimum pH towards Neutral

**Figure 5:**
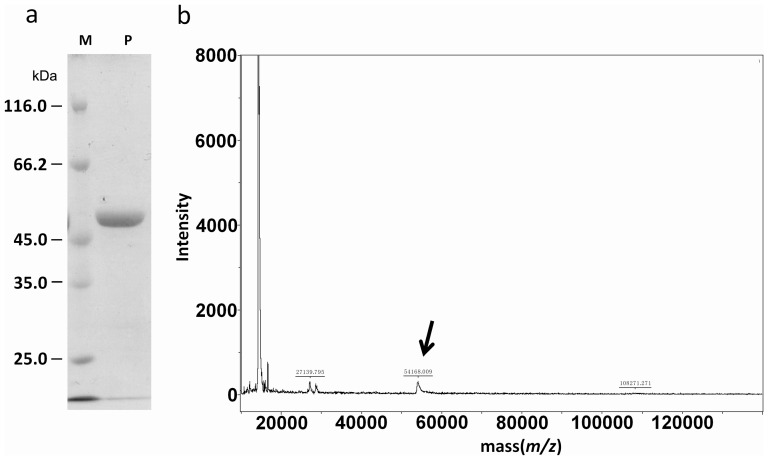
Analysis of purified recombinant M-inv2. (a) SDS–PAGE analysis of M-inv2 protein stained with coomassie blue. Lane M, markers for molecular size (kDa); Lane P, protein sample. The M-inv2 is at 54 kDa by SDS-PAGE. (b) MALDI MS analysis of recombinant M-inv2, the arrows indicate the target protein peaks.

Uninv2 has 120 amino acids at the N terminus that are not present in the invertase from *T. maritima*. This sequence contains a 20 amino acids signal peptide and a 100 amino acids domain of unknown function. PCR was used to delete amino acids 1–120 from uninv2. The PCR product was sequenced three times to confirm the deletion. The truncated protein was named M-inv2. M-inv2 was purified with Ni-NTA chromatography to give a single band by SDS-PAGE analysis (Fig. 5c) TOF-MS analysis showed the molecular weight as 54 kDa (Fig. 5d). The effects of pH on uninv2 and its truncated form, M-inv2, were compared. Interestingly, the loss of the N-terminal domain altered the optimum pH to a more neutral pH. M-inv2 had an optimum pH of 6.0 compared with pH 4.5 for uninv2. M-inv2 was active between pH 3.5 and 7.5, and retained 50% of its activity after incubation at pHs of between pH 4.5 and 6.5 (Fig. 3a). Thus, the deletion of the N-terminal domain of uninv2 shifted the enzyme’s optimum pH by 1.5 units towards neutral pH. Compared with uninv2, M-inv2 was found to be active over a narrower range of temperatures (25–55°C), and it retained 50% of its activity after incubation at temperatures of between 35°C and 47°C (Fig. 3b). However, the optimum temperature of the truncation did not change from that of wild type (45°C). The loss of the N-terminus did not affect the enzymatic activity of the protein against oligosaccharides.

A neutral pH optimum is valuable for industrial applications of invertases, such as in the production of invert sugars, and as enzyme electrodes for biosensors [7], [20]. To date, the most studied invertase was the acid invertase from *S. cerevisiae* which is active between pH 4–7 with an optimum activity at pH 5.0 [14]. Though, some neutral pH active invertases were reported [10], [12], [13], [21], there are no data about enzyme stability. The neutral invertases from plants typically have very low K_m_s to sucrose, but with slow hydrolysis speeds [22], [23]. M-inv2 showed a near neutral optimum pH and strong pH stability making it potentially useful as a biosensor.

### Kinetic Parameters of Uninv2 and Truncation

When uninv2 and M-inv2 were incubated with a range of sucrose concentrations, typical Michaelis–Menten kinetics were observed (Fig. S2). A V_max_ of 1302.2±16.6 µmol min^−1 ^mg^−1^, a K_m_ of 17.2±0. 7 mM and a k_cat_ of 1447.6 s^−1^were measured for uninv2. The V_max_, K_m_ and k_cat_ for M-inv2 were 961.7±36.04 µmol min^−1 ^mg^−1^, 32.5±2.4 mM and 868.2 s^−1^, respectively. Table 1 compares the properties of various invertases from a number of different sources. The enzyme with the highest activity is the invertase from *S. cerevisiae*, while the activity of uninv2 is lower than the invertase from *T. maritima* but is higher than the one from *Bifidobaterium adolescentis*. Uninv2 have a lower K_m_ compared with invertase from *S. cerevisiae* and *T. maritima*, thus its catalytic efficiency is higher than that of invertase from *T. maritima*. M-inv2 have higher K_m_ than uninv2 and its catalytic efficiency is lower than that of uninv2 and invertase from *T. maritima*. The neutral invertases from plants, such as *Beta vulgaris* and pea seeding, typically have very low K_m_s to sucrose, with slow hydrolysis speeds. Truncation of uninv2 results in a lower V_max_ comparing with uninv2, though it is still higher than that of the invertase from *B. adolescentis.*

**Table 1:** Kinetic parameters of invertases from various sources.

Sources	Optimum pH	K_m_ (mM)	V_max_ (µmol min^−1 ^mg^−1^)	k_cat_ (s^−1^)	k_cat_/K_m_ (mM^−1^s^−1^)
***B. adolescentis*** ** G1 ** [**21**]	5.7	38	79	NR	NR
***T. maritima*** **** [**18**]	5.5	64	3117	2600	40.6
***S. cerevisiae*** **** [**30**]	5.0	26.1	8230^a^	9400	360.2
***Rhodotorula glutinis*** **** [**3**]	4.5	227	0.096^b^	NR	NR
**Pea seeding ** [**31**]	4.0	4.41	8.41	NR	NR
***Hordeum vulgare*** **** [**22**]	5.5	12	80	NR	NR
***B. vulgaris*** **** [**32**]	8.0	0.7	19.6	NR	NR
***Vicia faba*** **** [**23**]	7.4	10.1	585.9	NR	NR
**Uninv2 (this study)**	4.5	17.8	1302.3	1447.6	81.3
**M-inv2 (this study)**	6	32.5	961.7	868.2	26.7

NR, not reported.

a The data come from reference [33].

b the unit was mmol min^−1^.

### pH Tolerance and Storage Stability of Uninv2 and M-inv2

**Figure 6:**
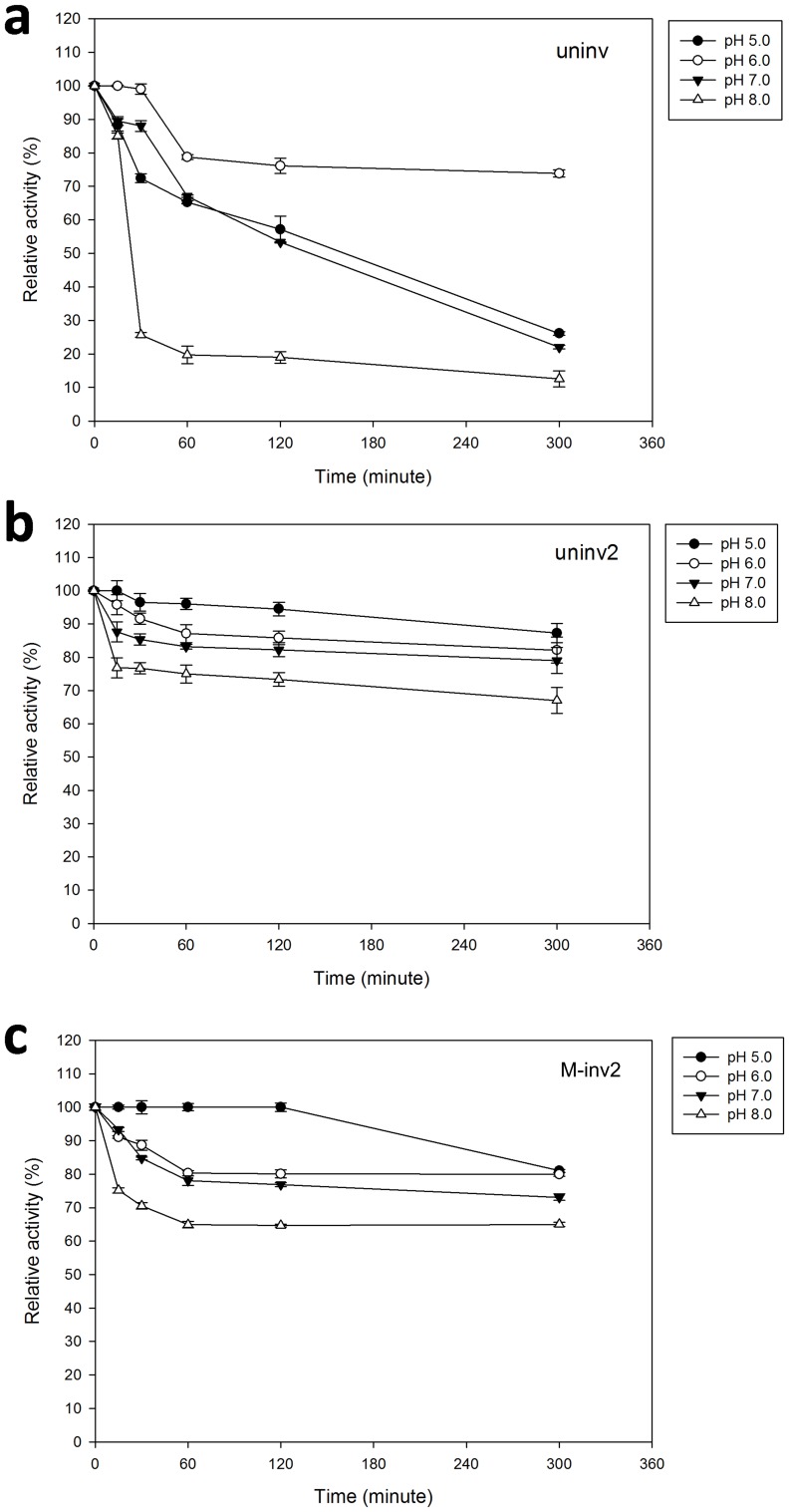
The pH stability of the enzyme at 37°C. The pH stability of uninv (a), uninv2 (b) and M-inv2 (c) were tested at 37°C. The protein was incubated in pH 5.0 (closed cycles), pH 6.0 (open cycles), pH 7.0 (closed triangles) and pH 8.0 (open triangles) buffer at 37°C and was tested for residual activity under the optimum conditions at different intervals. The error bars represent the standard deviation of triplicate measurements.

The pH stability of the enzyme at 37°C was compared between uninv [16], uninv2 and M-inv2 (Fig. 6). Uninv and uninv2 were found from the same metagenomic DNA library. They have a 30% identities and 43% positives in amino acid sequences [16]. Uninv was a neutral invertase with hydrolysis activity towards sucrose in the pH range of 6.0–8.0 with an optimum pH of 6.5. At 37°C, uninv was only stable at pH 6.0 (lost 26% activity). It was unstable at other pHs, especially at pH 8.0, where it lost 75% activity at 30 min and 87% at 300 min (Fig. 6a). Uninv2 and M-inv2 were very stable at all the tested pHs (Fig. 6b and 6c).

**Figure 7:**
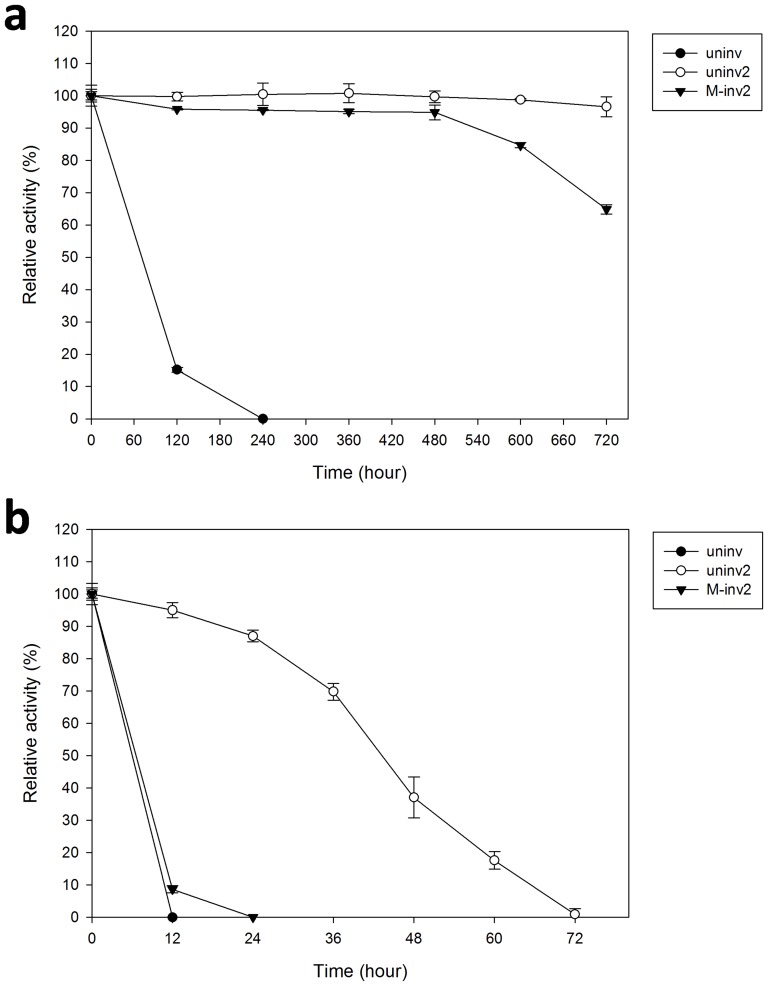
Storage stability at different temperatures. The storage stability of uninv (closed cycles), uninv2 (open cycles) and M-inv2 (closed triangles) was performed at 4°C (a) and 37°C (b). Proteins were incubated in buffer at pH 6.0 for uninv and pH 5.0 for uninv2 and M-inv2, the incubation temperatures were 4°C and 37°C. Aliquots were withdrawn for activity measurement under the optimum conditions at different intervals. The error bars represent the standard deviation of triplicate measurements.

The storage stabilities of uninv, uninv2 and M-inv2 were compared in Fig. 7. When stored at 4°C, uninv became in-active after 10 days. It is worthy to note that uninv2 still retained 97% activity after 30 days, and M-inv2 retained 65% activity (Fig. 7a). When stored at 37°C, uninv showed no activity after 12 h, M-inv2 lost its activity after 24 h, and uninv2 stood for a long time, it lost all activity after 72 h (Fig. 7b).

The widely used invertase from *S. cerevisiae* lost 35% activity after a 300 min incubation at pH 5.0, and lost about 70% activity after the same time at pH 6.0 [15]. At pHs greater than 7.0, it was not stable even for 4 h [24]. The pH-dependent activity of enzymes is set primarily by the pKa values of one or a few key ionizable groups within its active site cleft [25]. Thus, the factors that establish the precise pKa values of these catalytically essential groups are important. Goetz and Roitsch shifted 0.6 units of the pH optimum of an invertase to more basic values (from pH 3.8 to 4.4) by substitution of the proline residue with valine in the conserved motif of this invertase [26]. It was also observed that substitution of single amino acid on *Aspergillus kawachii* xylanase C elevated its pH optimum from pH 2 to 5 [27]. Joshi et al. (2000) concluded that family 11 xylanases had a special electrostatic interactions between acid/base catalyst (Glu 172) and the substituted position (Asp 35), the strong hydrogen bonding interaction stabilized the transition state for glycosyl transfer at acid pH condition [28]. It is different for immobilized invertase, some authors have reported an increase in optimum pH and broadening of the pH profile after immobilization [29], it is concluded that strong interactions between enzyme and support will affect the intra-molecular forces responsible for maintaining the conformation of the enzyme that would lead to a change in activity [15]. The ability of uninv2 to be stored for a long period of time with the minimal activity loss was superior to invertase from *S. cerevisiae*, which lost all activity over 15 days at 5°C [15].The ability to stand for neutral pH condition and the long storage stability of uninv2 make it ideal for use in a variety of industrial applications. Besides of the food and alcohol fermentation application, it is also a suitable enzyme used in an enzyme biosensor which need strong stability enzymes.

### Conclusions

Stability of an enzyme is important for its application in industry. In this study, invertase uninv2 with high pH tolerance and storage stability was reported. It has a broad pH tolerance, comparing with other invertases intolerant to neutral or alkaline environments. While deletion of its N-terminal 100 amino acids, uninv2 was truncated from an acid pH optimum invertase to a neutral pH optimum invertase, and the truncation still retained the high pH tolerance and storage stability of wild-type enzyme. These characteristics make them far superior to invertase from *S. cerevisiae*, which is a most used industrial enzyme. This suggests that uninv2 and its truncation have the potential for use in industry.

## Supporting Information

Figure S1 **Alignment of glycoside hydrolases from glycoside hydrolase family 32.** The alignment showed the most similar proteins with uninv2 in the BlastP analysis. The sequences were identified as follows: Bai: protein from *Bacteroides intestinalis* DSM 17393 (EDV04068), Blh: protein from *Blautia hansenii* (ZP_03547458), Cag: protein from *Capnocytophaga gingivalis* ATCC 33624 (EEK13630), Clb: protein from *Clostridium beijerinckii* (YP_001310947), Cls: protein from *Clostridium* sp. L2–50 (ZP_02075141), Coe: protein from *Coprococcus eutactus* (ZP_02205360), Pad: protein from *Parabacteroides distasonis* ATCC 8503 (ABR45076), Rug: protein from *Ruminococcus gnavus* (ZP_02040442), Spl: protein from *Spirosoma linguale* DSM 74 (EEP01562), Thm: protein form *Thermotoga maritima* (AAD36485), Thp: protein from *Thermoanaerobacter pseudethanolicus* (ABY94292), Ths: protein from *Thermoanaerobacter* sp.X514 (ABY92416). Accession numbers (GenBank or Swissprot) of the enzymes were shown in parenthesis after each original strain. The alignment was performed with MUSCLE and the figure was produced with BOXSHADE.
(TIF)

Figure S2 **Michaelis-Menten graph of uninv2 and M-inv2.** Reactions were carried out in the optimum conditions using 0.1µg enzyme. The substrate concentrations were 2.9, 8.7, 14.6, 29.2, 58.5 and 146.2 mM. The figure showed one set of data. Inset showed the Lineweaver-Burk plot.
(TIF)
