# Supplementary figures and images for: Characterization of an Invertase with pH Tolerance and Truncation of Its N-Terminal to Shift Optimum Activity toward Neutral pH
Source: PLoS One. 2013 Apr 19;8(4):e62306. doi: 10.1371/journal.pone.0062306 (PMC3631178; doi:10.1371/journal.pone.0062306)

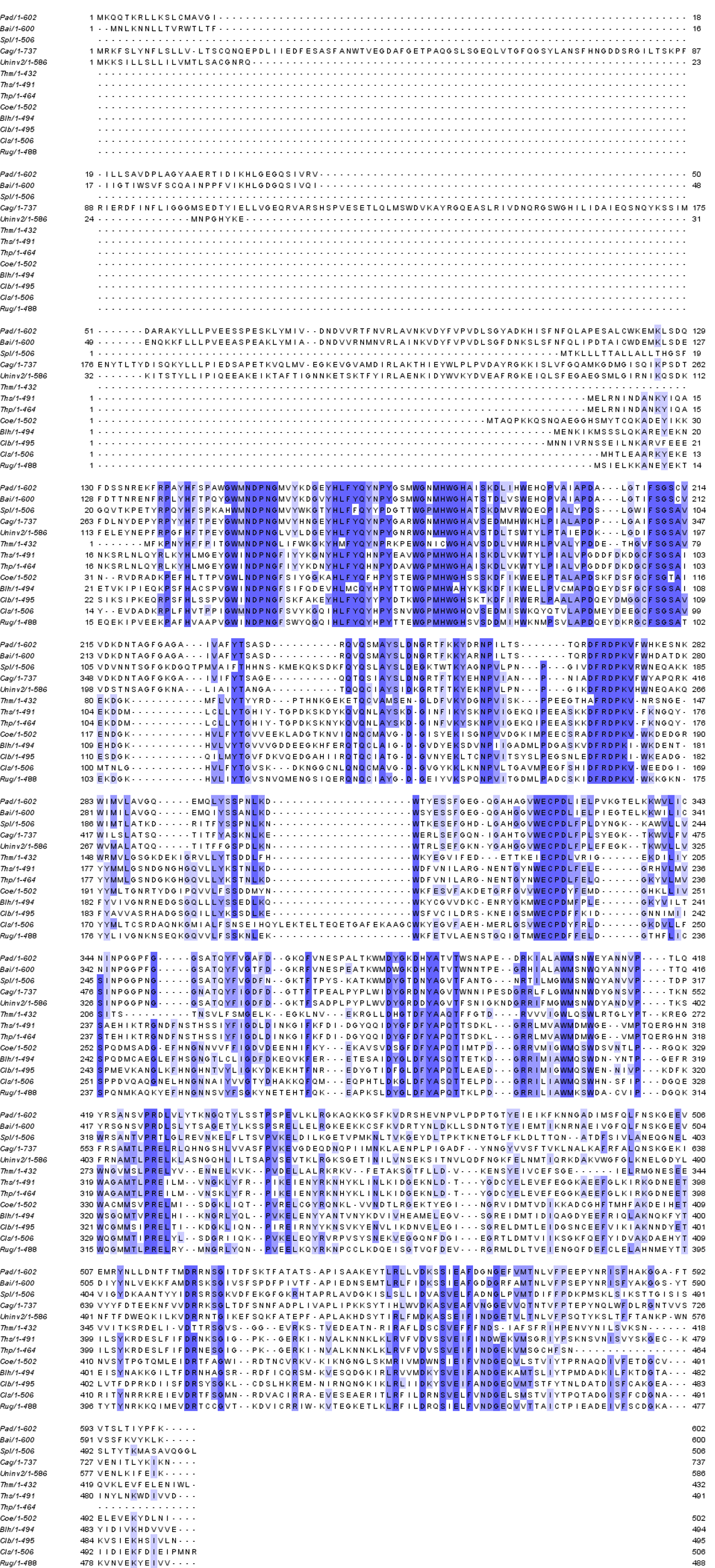

Supplement: Figure S1 — Alignment of glycoside hydrolases from glycoside hydrolase family 32. The alignment showed the most similar proteins with uninv2 in the BlastP analysis. The sequences were identified as follows: Bai: protein from Bacteroides intestinalis DSM 17393 (EDV04068), Blh: protein from Blautia hansenii (ZP_03547458), Cag: protein from Capnocytophaga gingivalis ATCC 33624 (EEK13630), Clb: protein from Clostridium beijerinckii (YP_001310947), Cls: protein from Clostridium sp. L2–50 (ZP_02075141), Coe: protein from Coprococcus eutactus (ZP_02205360), Pad: protein from Parabacteroides distasonis ATCC 8503 (ABR45076), Rug: protein from Ruminococcus gnavus (ZP_02040442), Spl: protein from Spirosoma linguale DSM 74 (EEP01562), Thm: protein form Thermotoga maritima (AAD36485), Thp: protein from Thermoanaerobacter pseudethanolicus (ABY94292), Ths: protein from Thermoanaerobacter sp.X514 (ABY92416). Accession numbers (GenBank or Swissprot) of the enzymes were shown in parenthesis after each original strain. The alignment was performed with MUSCLE and the figure was produced with BOXSHADE. (TIF) [file pone.0062306.s001.tif]
